# Comparative evaluation of Rheological characteristics of Giomers and other Nano-flowable resin composites *in vitro*

**DOI:** 10.1080/26415275.2021.1996239

**Published:** 2021-11-08

**Authors:** Loulwa M. Al-Saud

**Affiliations:** Division of Operative Dentistry, Department of Restorative Dental Sciences, College of Dentistry, King Saud University, Riyadh, Saudi Arabia

**Keywords:** Complex viscosity, flowable composite, giomers, rheology, viscoelasticity

## Abstract

**Objective:**

The purpose of this research was to determine the viscoelastic properties of a group of commercially available nano-flowable resin composites; and to explore the relation between these properties and the materials’ composition (with/without fluoride), filler size description (nano-filled, nanohybrid and submicron-filled) and filler loading (by volume).

**Methods:**

Rheological measurements were performed using a rheometer. A Dynamic frequency sweep test was conducted to evaluate the complex viscosity, storage and loss moduli, loss tangent, and complex shear modulus at an angular frequency (*ω*) of 0.1–100 rad/s. Comparative evaluations of the nano flowable resin composites on rheological properties was performed, and statistically analyzed using one-way ANOVA.

**Results:**

The results indicated that all the tested materials exhibited shear-thinning flow behaviour. As the shear rate increased, the complex viscosity of the nano-flowable composites decreased. The nanohybrid filled flowable resin composites exhibited the highest complex viscosity, while the nano-filled flowable resin composites exhibited the lowest value. The submicron-filled materials exhibited the lowest complex shear moduli and loss tangent values. **Conclusions:** The findings from the current study provided comprehensive evaluation of the rheological properties of different nano-flowable composites. The observed differences in rheological properties among the tested materials were independent of their fluoride content or filler size. Furthermore, no relationship was found between the complex viscosity of the tested nano-flowable resin composites and their filler volume.

## Introduction

Progressive advancement in filler technology has paved the path for the development of resin-based composites (RBCs), including flowable composites, with improved properties for a wide range of applications. Nanotechnology has facilitated the development of RBCs with nano-sized filler particles (<100 nm) which exhibited reduced dimension with a significantly increased surface area to volume ratio [1,2]. These nanofillers can be in the form of discrete particles (nanomeric particles) or agglomerated clusters of nanoparticles (nanoclusters) [3].

Nanotechnology has also facilitated more effective incorporation of antimicrobial agents such as fluoride into resin composites [4]. Fluoride is added to resin composites to enhance their anti-bacterial properties as they lack the ability to release fluoride naturally. Generally, fluoride can be incorporated into resin composite in various forms (e.g. organic fluoride, inorganic salts or leachable glasses) [5] using three different approaches including the addition and blending of water-soluble fluoride salts (e.g. NaF) with the polymer matrix; matrix-bound fluoride systems; or fluoride-releasing filler systems [6].

The mechanism and amount of fluoride release from fluoridated resin composite is a complex process with evident variations among different materials. This can be attributed to several factors such as the resin matrix formulations, silane treatment, material porosity, fluoridated filler type and particle size, all of which affect the ability of fluoride to leach from the composite resin [5,7,8].

Currently, nano-flowable resin composites with various filler size formulations are widely available for many clinical applications with excellent physical and mechanical properties [9]. Their low viscosity and high flowability offer better adaptation to the internal cavity walls, accessibility to the difficult-to-reach areas of the preparation and relative ease of application (e.g. material dispensing *via* fine-gauge needles).

The characteristic low viscosity of flowable composites is achieved by reducing the filler content (up to 37–53% by volume), increasing the ratio of the diluent monomers to enhance the fluidity, or by adding modifying agents, such as surfactants, to the resin matrix [10,11]. The viscosity of RBCs is significantly affected by the organic matrix, inorganic filler characteristics (size, shape and surface treatment), interlocking between the filler particles, and interfacial interaction between the resin matrix and filler particles [12].

Viscosity, which describes the material resistance to flow, is a complex property that can be assessed comprehensively using rheological techniques [13]. Rheology is the study of the flow behavior of materials (using a rheometer) to characterize their viscoelastic properties.

Several studies have been conducted to investigate the rheological properties of RBCs, including flowable resin composites [14–17]. Significant differences have been observed in the viscoelastic behavior of not only the different classes of resin composites (flowable, universal hybrid and packable composites), but also among various materials from the same class [14].

The shear rate, temperature variations, filler and resin monomer formulations significantly impact the rheological and flow properties of RBCs [12,15]. In addition, the filler particle size and distribution significantly affect the viscosity and packing stress of different RBCs, thus affecting their handling characteristics [16]. Recently, it has been reported that the filler type (e.g. spherical particles or rod fibres) considerably affects the viscoelastic properties of RBCs [17].

Although existing research has illuminated the influence of some parameters on the flow behavior and the viscoelastic properties of nano-flowable RBCs, no study to date has examined the differences in rheology among various nano-flowable resin composites based on their fluoride content. The mechanism of fluoride release (both immediately and on the long-term) is complex and multifactorial. It can affect the resin matrix as well as the resin filler interface, all of which may possibly influence the viscolelastic behavior of the material and other properties. In-depth evaluation studies on the rheology of flowable resin composites with various formulations are needed to facilitate an informed selection and manipulation of these materials clinically.

Therefore, this study was set out to examine the rheological behavior and viscoelastic properties of eight commercially available nano-flowable resin composites with and without fluoride release.

Two null hypotheses were proposed. First, there were no significant differences between the rheological properties of the tested materials depending on their fluoride content or filler size. Second, there was no correlation between the filler loading (by volume) and the viscosity of the tested materials.

## Materials and methods

This *in vitro* study was registered at the College of Dentistry Research centre (project registration # FR 0491).

Eight commercially available resin-based nano-flowable composites (with and without fluoride) were investigated in the current study. Their composition, classification and technical profiles are presented in Table 1. For brevity, the abbreviated codes will be used to refer to the tested materials throughout the paper instead of the full name.

**Table 1.** Nano flowable resin composite materials evaluated in the current study.

Type	Code	Flowable Composite	Manufacturer	Lot no.	Classification	Resin Matrix	Filler loading		Filler size range
							(vol%)	(wt%)	
Non-Fluoridated flowable resin composite	FK	Filtek^TM^ Z350 XT	3M^TM^ ESPE^TM^	N951675	Nano-filled Flowable	Bis-GMA^a^, TEGDMA^a^ & Procrylat resins	46%	65%	0.1–5 μm
	FF	FUSION FLO	PrevestDenPro**^®^**	2291717	Nano Hybrid Flowable	Bis-GMA, Ethoxlated bis-DMA^a^, UDMA^a^ TEGDMA	Total inorganic filler 70%.		NA
	NF	Nexcomp Flow	Meta Biomed Co. LTD.	1808272	Nano Hybrid Flowable	Bis-GMA, UDMA, Bis-EMA^a^, TMPTMA^a^	37 %	NA	0.04–0.7 μm
	EFQ	Estelite^®^ FlowQuick	Tokuyama Dental Corp.	183E48	Spherical Submicron filled flowable *(supra-nano spherical filler)*	Bis-MPEPP^a^ , TEGDMA, UDMA	53 %	71 %	0.04–0.6 μm
	EHF	Estelite^®^ FlowQuick High flow	Tokuyama Dental Corp.	134E07		Bis-GMA, TEGDMA	49%	68%	
Fluoride-releasing flowable resin composite	B00	Beautifil flow Plus F00 (Zero flow)	Shofu Dental	061767	Fluoride-releasing Nano-hybrid flowable (Giomer)	Bis-GMA, TEGDMA	47%	67.3%	0.01–4.0 μm
	B03	Beautifil flow Plus F03 (low flow)	Shofu Dental	111887		Bis-GMA, TEGDMA	46.3%	66.8%	0.01–4.0 μm
	WV	Wave	SDI Limited	180634	Fluoride-releasing Nano-filled Flowable	UDMA	41%	65%	0.02 − 10 μm

^a^Bis-GMA: bisphenylglycidyl dimethacrylate; TEGDMA: triethylenglycol dimethacrylate; Bis-EMA: bisphenol A ethoxylated dimethacrylate; UDMA: urethanedimethacrylate; TMPTMA: Trimethylolpropane trimethacrylate; Ethoxlated bis-DMA: Ethoxlated Bisphenol A Dimethacrylate; Bis-MPEPP: Bisphenol A polyethoxy methacrylate.

### Rheological measurements

In the current study, a Modular Compact Rheometer (MCR 72, Anton Paar GmbH, Graz, Austria) was used to perform the rheological assessment for all flowable composites tested. The parallel-plate geometry PP 25 (diameter of 25 mm) was employed for viscosity measurements using the dynamic oscillatory shear tests. The gap size between the plates was 250 μm, at a controlled temperature of 25 °C using the Peltier-controlled Plate System (CoolPeltier^TM^).

The flowable resin composite paste was applied at the centre of the stationary lower plate, after which the upper plate was moved and lowered to reach the specified gap. Before starting each measurement, the excess composite material was carefully trimmed from around the plate with spatula (so that the paste does not extrude beyond the external diameter of the upper plate). After the completion of each test, the upper plate was removed and cleaned.

Dynamic frequency sweep applies a sinusoidal strain of constant peak amplitude over the selected frequency range. This test was performed for all tested materials to evaluate complex viscosity (η*), storage (elastic) shear modulus (G’), loss (viscous) shear modulus (G”), loss tangent (tan *δ*), and complex shear modulus (G*) as a function of angular frequency (*ω*) = 0.1–100 rad/s. Definitions of the evaluated rheological properties are presented in Table 2. Three replicate measurements were performed for each material.

**Table 2.** The rheological properties evaluated in the current study.

Rheological properties	Symbol	Unit	Definition
Complex viscosity	η*	Pa.s	The viscoelastic flow resistance of the material for practical applications. It describes the internal friction of a material under oscillatory shearing stresses
Storage modulus	G’	Pa	The elastic portion of the viscoelastic behavior, which describes the solid-state behavior of the sample (i.e. measure of the stored energy)
Loss modulus	G’’	Pa	The viscous portion of the viscoelastic behavior, which describes the liquid-state behavior of the sample (i.e. measure of the energy lost as heat)
Loss tangent/ Loss factor	tan *δ*	–	The ratio of the two portions of the viscoelastic behavior.
Complex shear Modulus	G*	Pa	Describes the entire viscoelastic behavior of a sample

Prior to the frequency sweep test, an amplitude sweep test was performed to examine the strain range of the uniform output signal. This is in order to perform all the subsequent measurements within each material’s linear viscoelastic range of deformation.

### Statistical analysis

The rheological tests outputs were calculated using the rheometer software (RheoCompass™). Independent-samples t-test was run to determine if there were any differences between the two categories of tested materials (fluoridated/non-fluoridated) for each rheological measurement individually (i.e. complex viscosity, storage modulus, loss modulus, loss factor, and complex shear modulus).

One-way ANOVA with Bonferroni correction was run to investigate whether the rheological properties of the tested materials differ based on their filler size (nano filled, nano hybrid or submicron filled) as reported by the manufacturers.

Additionally, a Pearson’s product-moment correlation was run to assess the relationship between complex viscosity and filler loading (by vol%) of all tested materials. FusionFlu was excluded from the correlation analysis since there was no available information about the filler loading by volume from the manufacturer.

Data were analyzed using a statistical software (SPSS v.20, IBM Corp, Armonk, NY) at (*p* < .05) significance level.

## Results

Preliminary analysis revealed that the data were normally distributed for each group, as assessed by Shapiro-Wilk test (*p* > .05) and there was homogeneity of variances, as assessed by Levene’s test for equality of variances (*p* = .577).

### Complex viscosity (η*)

The complex viscosity of all the tested flowable composites decreased with an increase in the frequency (shear rate), demonstrating the pseudoplasticity of the composites. The complex viscosity values at low-, high- and mid- range frequencies are listed in Table 3.

**Table 3.** Mean *(SD)* values for Complex viscosity (η*) in [Pa.s] of all tested materials as a function of frequency (*ω*) rad/s, at 25 ^o^C.

Nano flowable composite	Frequency ranges (rad/s)			Total Mean
	Complex viscosity Mean (SD)			
	Low	Mid	High	
	(*ω* = 0.1–0.631 rad/s)	(*ω* = 1–10 rad/s)	(*ω* = 15.8–100 rad/s)	(*ω* = 0.1–100 rad/s)
Filtek^TM^ Z350 XT	791.2 (550.8)	113.2 (42.9)	60.7 (4.9)	351 (475.5)
FUSION FLO	494.7 (300.7)	60.5 (30.5)	21.5 (1.8)	211.1(286.5)
Nexcomp Flow	965 (700.6)	147.8 (49.9)	73.8 (11.4)	431.1 (589.6)
Estelite^®^ FlowQuick	1417.5 (1029.7)	155.2 (85.5)	34.4 (9.02)	590.8 (891.7)
Estelite^®^ High flow	283.5 (155)	46.3 (21.5)	14 (1.9)	125.1 (156.1)
Beautifil flow Plus F00	2362.7 (1691.7)	302 (139.3)	83.5 (26.9)	1006.5 (1464.4)
Beautifil flow Plus F03	831.8 (613.4)	91.3 (47.4)	29.9 (2.5)	349.8 (524.7)
Wave	297.2 (206.8)	31.8 (17.5)	9.5 (0.67)	124.4 (183.1)

**Figure 1.**
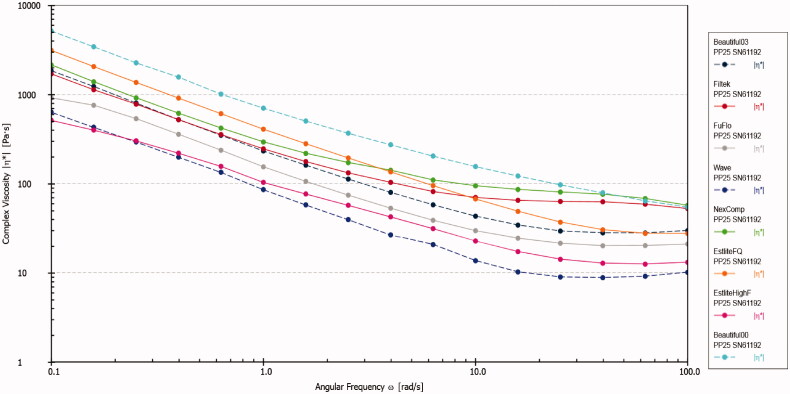
The complex viscosity (η*) in Pa.s of all tested materials as a function of frequency (*ω*) rad/s at 25 °C. The dotted lines represent fluoride releasing nano flowable composites, and the solid lines represent the non-fluoridated flowable composites.

Among all the composites investigated, B00 exhibited the highest viscosity values at all the frequencies, whereas Wv exhibited the lowest values (Figure 1).

**Figure 2.**
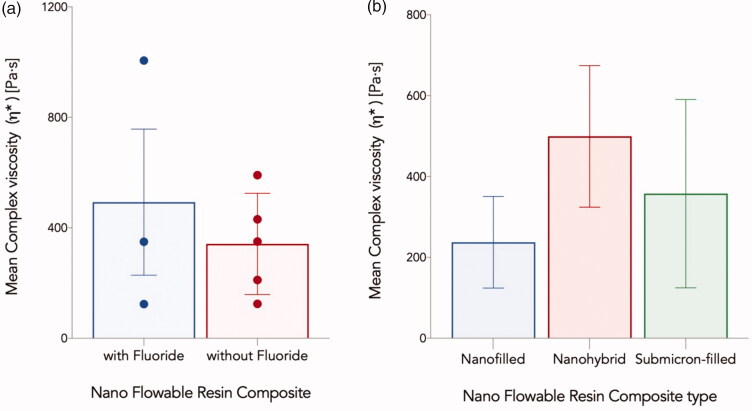
Mean complex viscosity η* in Pa.s for nano flowable resin composites investigated in the current study grouped based on: (a) fluoride content, and (b) Filler size description. Error bars represent the standard error of the mean.

The difference in the complex viscosity values of the fluoridated and non-fluoridated flowable composites (Figure 2(a)) was not statistically significant *(M* = 151.7, 95% CI [−391.4, 694.8], *t*(6) = 0.684, *p* = .52).

Similarly, the differences in the complex viscosity values among different nano-flowable composite groups (nanohybrid, submicron-filled, and nano-filled) (Figure 2(b)) were not statistically significant (*F* (2,5) =0.478, *p* = .646).

A weak correlation (based on Cohen’s classification)[18] between the complex viscosity and filler loading (by vol%), was observed; however, it was not significant; *r*(5) = 0.24, *p* = .608, (*r^2^* =0.056), with the filler loading (by vol%) explaining only 5.76% of the variation in the complex viscosity.

### Storage (G’) and loss (G”) moduli

**Figure 3.**
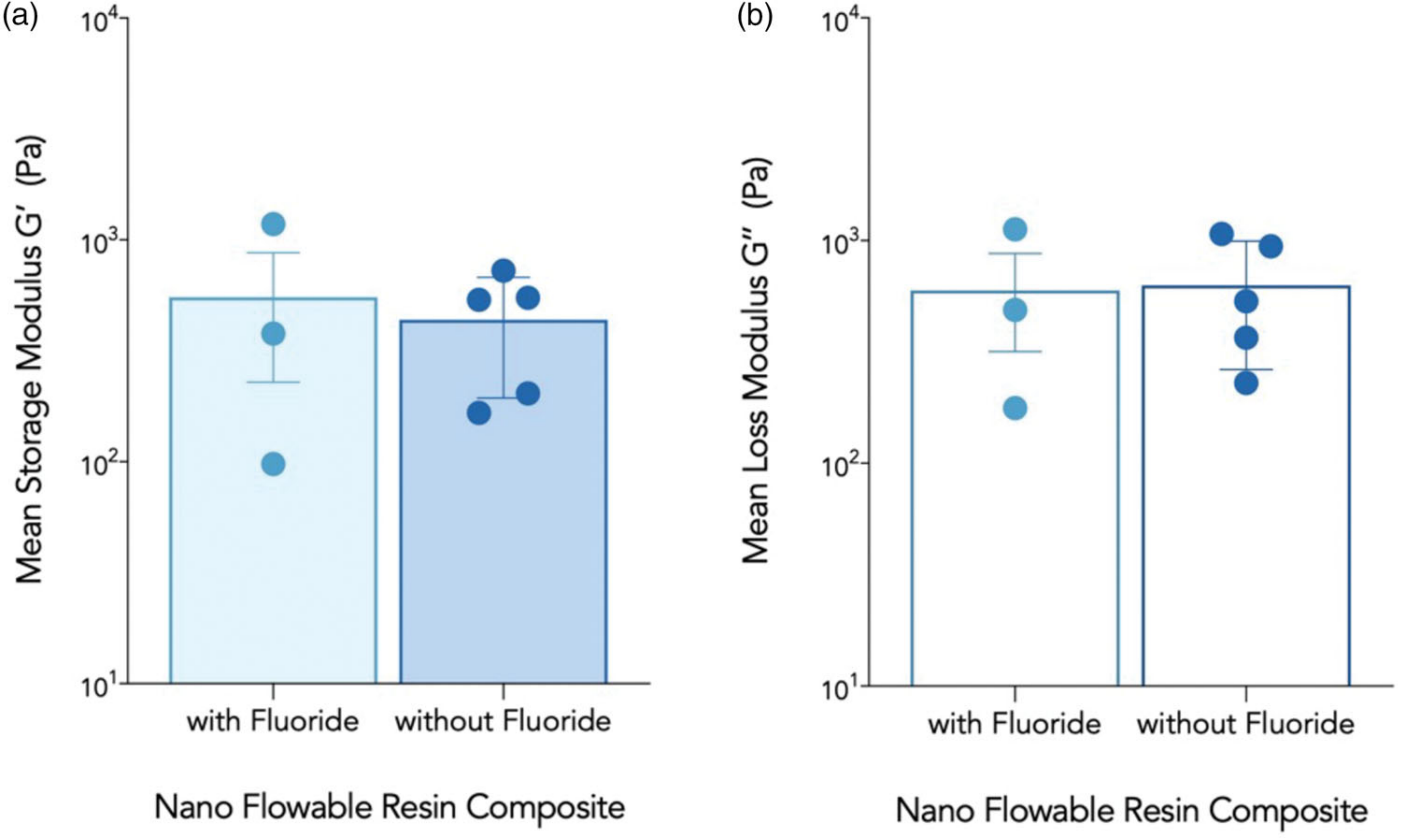
(a) Mean storage moduli (G’) in Pa for both groups of flowable composite. (b) Mean loss moduli (G”) in Pa for both groups of flowable composites. Error bars represent the standard error of the mean.

The storage and loss moduli of all the tested flowable composites increased with an increase in the frequency. The Differences between fluoridated and non-fluoridated flowable composites observed in each test were not statistically significant (*p* > .05) (Figure 3).

**Figure 4.**
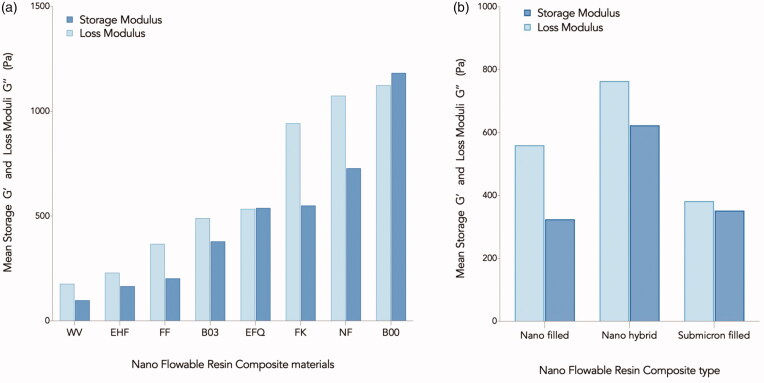
Mean Storage (G’) and Loss Moduli (G’’) in Pa, (a) for each tested nano flowable resin composite, and (b) for each category of flowable composite investigated in the current study.

Among all the composites investigated, B00 exhibited the highest storage and loss moduli, regardless of the frequency, whereas Wv exhibited the lowest storage moduli at all the frequencies (Figure 4(a)). The difference in the storage and loss moduli among different nano-flowable composite groups (nanohybrid, submicron-filled, and nano-filled) (Figure 4(b)) were not significant statistically (*F*(2,5) =0.557, *p* = .605).

For the loss moduli values, the assumption of the homogeneity of variances was violated, as assessed by Levene’s test for equality of variances (*p* = .005), therefore the Welch’s ANOVA was used here. The differences among the three groups were not statistically significant (Welch’s *F* (2,2.299) =0.924, *p* = .508).

### *Loss factor (tan* δ*)*

**Figure 5.**
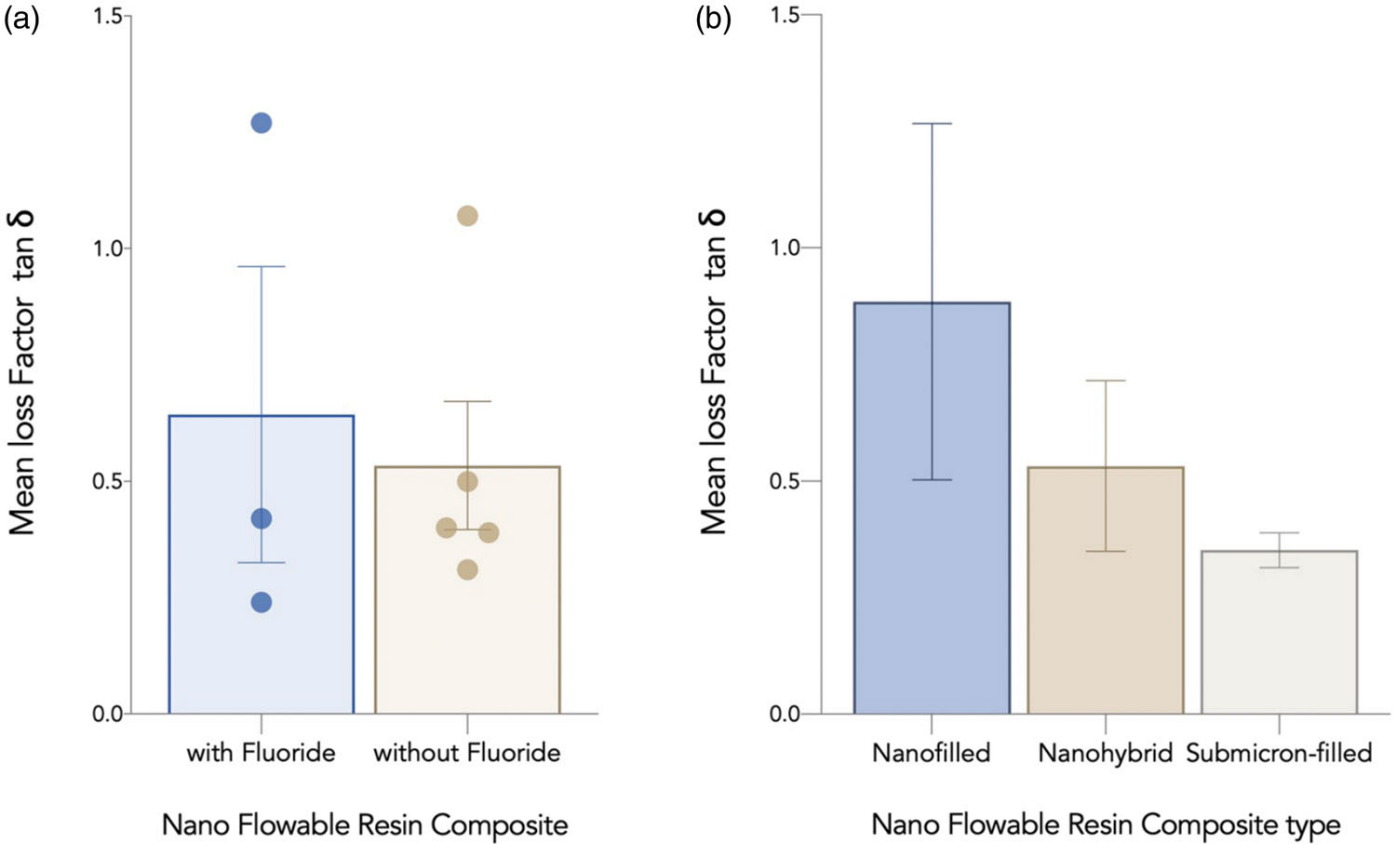
Mean Loss Factor (tan *δ*) for nano flowable composites investigated in the current study grouped based on: (a) fluoride content, and (b) Filler size description. Error bars represent the standard error of the mean.

The loss factor of all the tested materials increased as the frequency increased. At all the frequencies, Wv exhibited the highest loss factor, whereas B00 exhibited the lowest loss factor (comparable values to that of EFQ). The Differences in loss factor among different flowable composite groups (fluoridated/non fluoridated and Nanohybrid, submicron filled, and nano filled) were not statistically significant (*p* > .05) (Figure 5).

### Complex shear modulus G*

**Figure 6.**
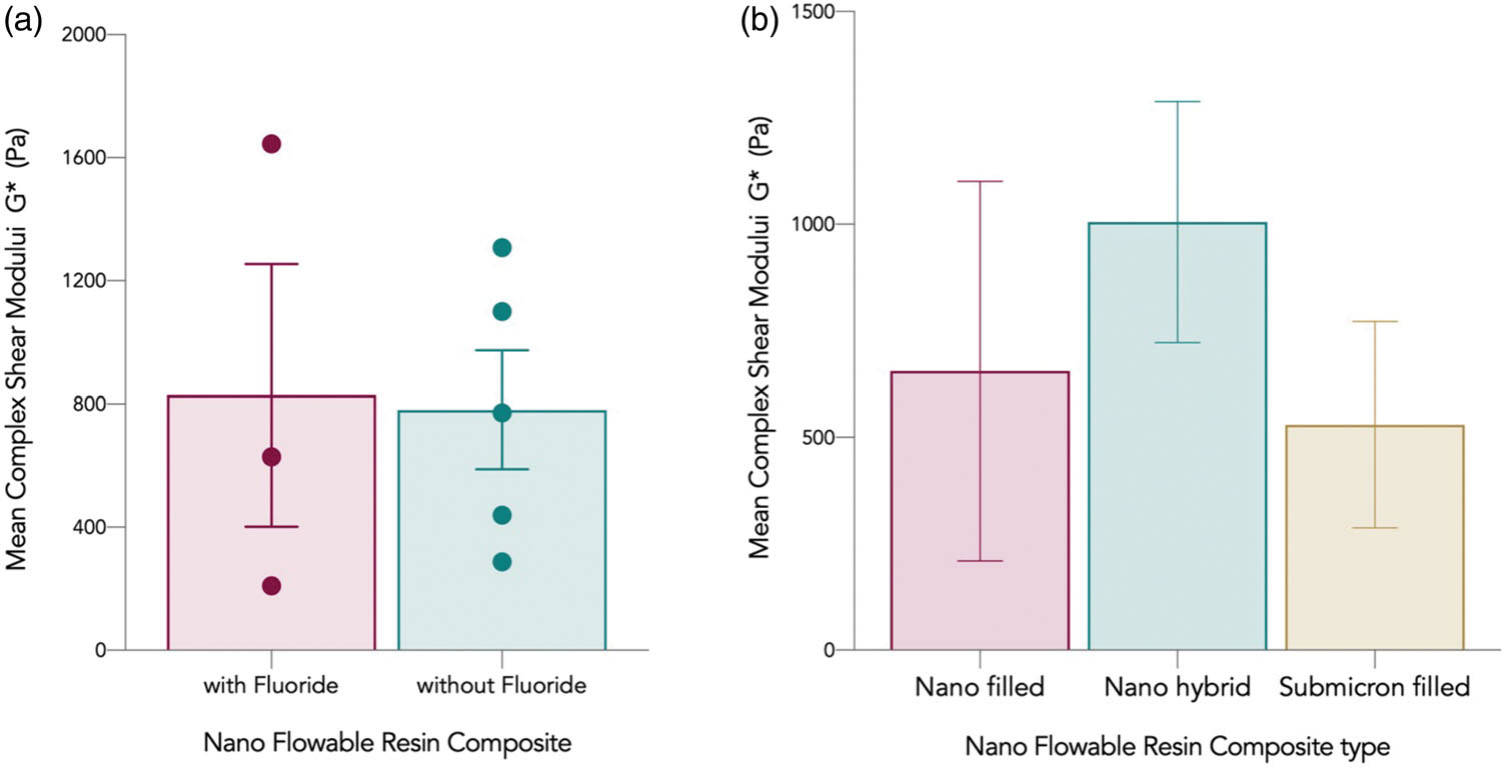
Mean complex shear moduli G* in Pa for nano flowable composites investigated in the current study grouped based on: (a) with and without fluoride, and (b) Filler size description. Error bars represent the standard error of the mean.

All tested materials exhibited an increase in the complex modulus from low to high frequency. The differences in complex shear moduli among different flowable composite groups (fluoridated/non fluoridated and Nanohybrid, submicron filled, and nano filled) were not statistically significant (*p* > .05) (Figure 6).

**Figure 7.**
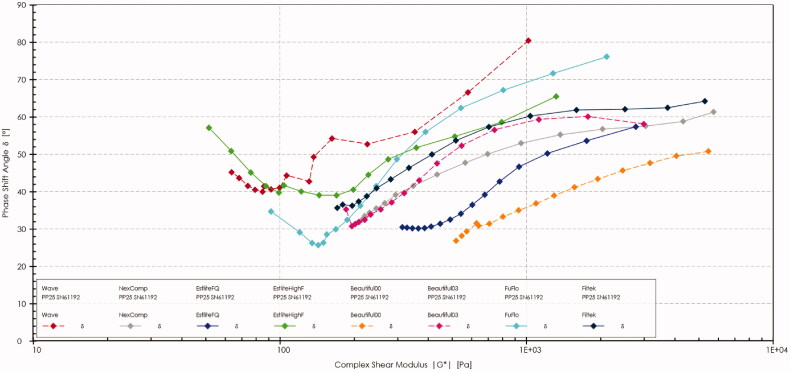
Van Gurp-Palmen plot (vGP) of all tested nano flowable composite materials. The dotted lines represent fluoride releasing flowable composites, and the solid lines represent the non-fluoridated flowable composites.

The phase angle δ for each nano flowable resin composite tested was plotted against the corresponding absolute value of the complex shear modulus |G*|. This plot is known as the Van Gurp-Palmen plot (Figure 7).

## Discussion

In this study, a modular compact rheometer was used to examine the linear viscoelastic properties of eight nano-flowable resin composites. The selected parallel plates geometry is advocated to study the flow behavior of filled resin materials [13].

All the tested nano-flowable resin composites exhibited a shear-thinning non-Newtonian flow behavior (i.e. gradual decrease in the viscosity as the shear rate and frequency increase). This finding is in agreement with those reported previously for different types of resin-based composites [14,15,19] including fibre-reinforced resin composites [17]. Most filled materials typically exhibit non-Newtonian flow behavior [20], which can be attributed either to the mode of the filler particles alignment in the direction of the flow or to the shear-thinning behavior of the resin matrix itself [21].

Despite the minimal viscosity difference between Wv and EHF, their resin matrix compositions and filler volume are different. The filler volume of Wv was lower than that of EHF (41%, and 49%, respectively). The resin matrix of Wv is based on (UDMA), which is known to exhibit a significantly lower viscosity than (bis-GMA) monomer [22]. In contrast, the EHF resin matrix is based on bis-GMA (diluted in the less viscous TEGDMA monomer) while incorporating the novel Radical-Amplified Photopolymerization initiator technology (RAP technology™). Therefore, the low viscosity of Wv can be attributed to the composition of its resin matrix.

Although the most filled flowable composite (vol%) in this study was EFQ (53%) followed by EHF (49%), B00 exhibited the highest viscosity of 1006.5 Pa.s, which is considerably higher (by approximately 882 Pa.s) than the lowest viscosity values observed for Wv.

Both B00 and B03 are Giomers, based on surface pre-reacted glass (S-PRG) fillers. In the presence of water, the acid-reactive fluorosilicate glass is reacted with polyacids, freeze-dried, milled, silanized, ground, and used as fillers [23]. The hydrogel layer of the SPRG particles contains fluoride complexes which is an added source for fluoride release [24]. This filler types along with the aqueous oral environment and the continuous dissolution of water result in more water absorption and possible effects of water molecules on the materials internal structure (e.g. micro voids in the resin matrix, plasticization, or filler debonding) resulting in degradation or softening of the composite which may impact some physical and mechanical properties including hardness and viscosity [25]. Therefore, this type of filler content may have affected the flow behaviour of B00; however, this assumption requires empirical testing.

The low-frequency range viscosities simulate the material behavior at rest (0.1 rad/s); whereas the mid-frequency range viscosities (1–10 rad/s) relatively simulates the stage of material manipulation [14,26] (e.g. clinical application stage of flowable composite). According to Lee et al.[14], viscosity measurements at *ω* = 10 rad/s are the most clinically relevant values that can be used as basis for comparison. In this study, at the frequency of 10 rad/s, only a slight change was observed in the complex viscosity ranking among the tested materials.

The finding for the complex viscosity and filler volume fraction is consistent with that reported by Lee et al. [14]. The use of the reported vol % for comparison is based on the fact that the rheological properties of nano-flowable resin composites are more dependent on the filler particle surface area upon which the hydrodynamic forces act than on the filler particle density or weight% [14]. A limitation of the current study is that the inorganic filler volume percentages used in the analysis was not measured directly, rather it was based on the values reported by the manufacturers. Thus, it is recommended that future studies overcome this limitation by measuring the filler volume of the examined materials.

This finding demonstrates the effect of only one filler characteristic (volume fraction) on the viscosity of nano-flowable composites; however, other filler characteristics such as type, size, shape, salinization, and distribution may also affect the viscosity levels of resin composites [17]. Particle size distribution (PSD) is an important parameter that affects the viscosity of composite materials. Changes to PSD (i.e. narrowing or widening the distribution) is a known method for controlling viscosity [21]. Additionally, the mechanisms by which the filler particles interact with various resin matrix ratios and components is another important factor to be considered [14,15,26,27]. For example, when the fillers are insufficiently coated with silane coupling agent, the resin composite viscosity increased resulting in weak bonding to the matrix and uneven dispersion of the fillers within the matrix [28]. The silane interphase linking the matrix to the fillers is critical to the resin composite properties, its effect is even more pronounced in nanocomposites because of the characteristically large surface area per unit mass exhibited by the nanoparticle fillers [29].

The distinct effect of B00 (the most viscous material tested here) on the overall results of the comparative rheological evaluation was noteworthy. In other words, the higher complex viscosity, complex shear moduli and loss tangent values of the fluoride group could be attributed to the presence of B00 (this was evident when the test was run excluding the B00 values), although the B00 values were not outliers in the original preliminary normality testing. However, there is no evidence to suggest that the fluoride release properties of these materials may have contributed to the observed higher values. Further investigation is warranted with other types of fluoridated flowable composites.

In an attempt to understand whether the rheological properties of the tested flowable composites differed depending on the material filler size, the materials were further categorised as nano-filled, nanohybrid and submicron-filled. By examining the filler size range reported by the manufacturers, it was evident that all the materials could be described as nanohybrids; because the filler size range started at the lower end at (0.1–100 nm) and extended at the higher end of the range to sizes over 100 nm. According to Jandt and Watts, virtually all resin nanocomposites materials are nanohybrids encompassing micron sized or non-nano, submicron sized filler particles [30]. Moreover, the reported size range reflects the variations in material classification among manufacturers, as well as the diverse material composition and manufacturing formulations, despite being all classified as nano-flowable resin composites.

If the applied stress at various frequencies is large enough to disrupt internal microstructure of the material, the loss modulus (G”) will be higher than the storage modulus (G’) and the material flows (i.e. reaches its yield point). Conversely, if the applied stress was less than the material’s internal forces, the G’ value will be higher and the material would be able to return, at least partially, to its original structure, after deformation [31]. Clinically, this is seen as a thicker more cohesive flow of the material (as in B00), hence its indication for proximal wall build-up and its description as ‘sculptable’ (as per manufacturer).

In the current study, the increase in the storage modulus (G’) of the composites with an increase in the frequency indicates the dominance of their elastic behaviour and a reduction in the viscous behaviour, as indicated by the concomitant decrease in their complex viscosity values. The results obtained for Wv were consistent with those reported by Petrovic et al. [32].

The Van Gurp-Palmen plots of the tested materials suggested two patterns of their rheological behavior. From the high to mid G* values, the phase angle of (EHF, FF, Wv, and B03) dropped to a certain point (i.e. inflection point) [33] after which it increased again as it approached the limiting value of the phase angle (i.e. 90°). The other pattern was observed for (FK, NC, EFQ, and B00), which exhibited a continuous gradual increase in the phase angle from the low to high G* values. The highest phase angle was observed for Wv (approximately 80°) (i.e. higher elasticity and higher flow), while the lowest was observed for B00 (50°) (i.e. more viscous behavior at rest); all within the phase angle range of viscoelastic materials (*δ* = 0°– 90°).

The results obtained in this study provides new insights into the effect of certain parameters on the viscosity and flow behavior of different nano-flowable resin composites used in dentistry. It also highlights the complex nature of viscosity as handling property of dental resin composites. Future studies should explore other variables affecting viscosity and flow behavior of flowable resin composites (e.g. monomer type, filler particle shape, and filler size distribution). Diversity in rheological properties of nano flowable composites reflect the wide choices available to clinicians. Thus, careful selection of the type and viscosity of flowable composite that suites each clinical case is highly recommended and should be case-specific.

## Conclusion

Based on the result of this study, both null hypotheses were accepted, and it can be concluded that the differences in rheological properties and flow behaviors among the tested nano-flowable resin composites based on various parameters (filler size, volume, or fluoride content) were not statistically significant. Additionally, no relationship was found between the complex viscosity and filler loading (by vol%) of the tested nano-flowable resin composites.

## Data Availability

Any data that support the findings of this study are included within the article.
